# 2-{1-[2-(Bis{2-[1-(5-chloro-2-hydroxy­phen­yl)ethyl­ideneamino]eth­yl}amino)­ethyl­iminio]eth­yl}-4-chloro­phenolate toluene hemisolvate

**DOI:** 10.1107/S1600536809002906

**Published:** 2009-01-28

**Authors:** See Mun Lee, Hapipah Mohd. Ali, Kong Mun Lo, Seik Weng Ng

**Affiliations:** a Department of Chemistry, University of Malaya, 50603 Kuala Lumpur, Malaysia

## Abstract

In the toluene hemisolvated tripodal tris­(2-amino­ethyl)amine Schiff base, C_30_H_33_Cl_3_N_4_O_3_·0.5C_7_H_8_, one of the three imino N atoms is protonated, forming a hydrogen bond with the O atom at an adjacent benzene ring. The other two imino N atoms act as hydrogen-bond acceptors from phenolate OH groups. The toluene solvent mol­ecule is disordered about a centre of inversion.

## Related literature

For the unsolvated tris­{2-[(5-chloro­salicyl­idene)amino]eth­yl}amine, which is refined as a neutral mol­ecule, see: Kanesato *et al.* (2000 ▶).
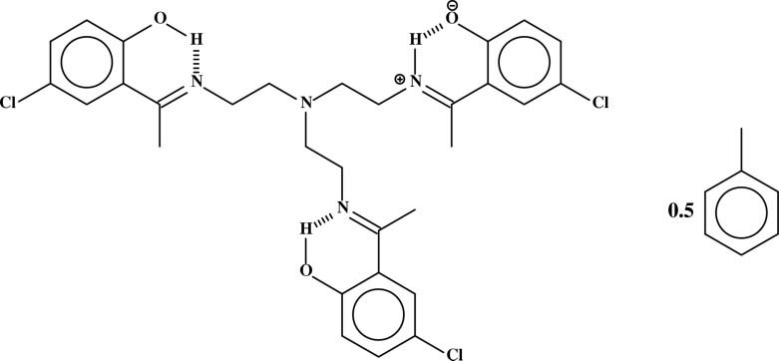


## Experimental

### 

#### Crystal data

C_30_H_33_Cl_3_N_4_O_3_·0.5C_7_H_8_
*M* *_r_* = 650.02
Triclinic, 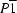

*a* = 7.3651 (2) Å
*b* = 11.1839 (2) Å
*c* = 20.1594 (5) Å
α = 100.618 (2)°
β = 97.765 (2)°
γ = 98.560 (2)°
*V* = 1591.20 (7) Å^3^
*Z* = 2
Mo *K*α radiation
μ = 0.33 mm^−1^
*T* = 100 (2) K
0.22 × 0.18 × 0.02 mm

#### Data collection

Bruker SMART APEX diffractometer
Absorption correction: multi-scan (*SADABS*; Sheldrick, 1996 ▶) *T*_min_ = 0.931, *T*_max_ = 0.993
14959 measured reflections
7269 independent reflections
4901 reflections with *I* > 2σ(*I*)
*R*_int_ = 0.033

#### Refinement

*R*[*F* ^2^ > 2σ(*F* ^2^)] = 0.050
*wR*(*F* ^2^) = 0.150
*S* = 1.04
7269 reflections
410 parameters
30 restraints
H atoms treated by a mixture of independent and constrained refinement
Δρ_max_ = 0.79 e Å^−3^
Δρ_min_ = −0.80 e Å^−3^

### 

Data collection: *APEX2* (Bruker, 2007 ▶); cell refinement: *APEX2*; data reduction: *SAINT* (Bruker, 2007 ▶); program(s) used to solve structure: *SHELXS97* (Sheldrick, 2008 ▶); program(s) used to refine structure: *SHELXL97* (Sheldrick, 2008 ▶); molecular graphics: *X-SEED* (Barbour, 2001 ▶); software used to prepare material for publication: *publCIF* (Westrip, 2009 ▶).

## Supplementary Material

Crystal structure: contains datablocks global, I. DOI: 10.1107/S1600536809002906/bt2855sup1.cif

Structure factors: contains datablocks I. DOI: 10.1107/S1600536809002906/bt2855Isup2.hkl

Additional supplementary materials:  crystallographic information; 3D view; checkCIF report

## Figures and Tables

**Table 1:** Hydrogen-bond geometry (Å, °)

*D*—H⋯*A*	*D*—H	H⋯*A*	*D*⋯*A*	*D*—H⋯*A*
O1—H1O⋯N1	0.85 (1)	1.69 (2)	2.486 (3)	155 (5)
O2—H2O⋯N2	0.85 (1)	1.70 (2)	2.507 (3)	158 (4)
N3—H3N⋯O3	0.89 (1)	1.62 (2)	2.474 (3)	158 (5)

**Fig. 1.**
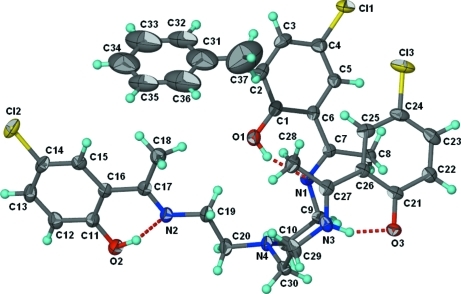
Anisotropic displacement plot (Barbour, 2001) of C30H33Cl3N4O3.0.5C7H8 at the 70% probability level. Hydrogen atoms are drawn as spheres of arbitrary radius.

## supplementary crystallographic information

### Experimental

Tris(2-aminoethyl)amine (1.46 g m 10 mmol) was condensed with 5-chloro-2-hydroxyacetophenone (5.12 g, 30 mol) in refluxing ethanol (100 ml) to yield the Schiff base. The solvent was removed and the product recrystallized from toluene.

### Refinement

Carbon-bound H-atoms were placed in calculated positions (C–H 0.93 to 0.99 Å) and were included in the refinement in the riding model approximation, with *U*(H) set to 1.2 to 1.5*U*(C). The methyl H-atoms were rotated to fit the electron density.

The toluene molecule is disordered about a center-of-inversion; the aromatic ring was refined as a rigid hexagon of 0.5 site occupancy. The 1,2-related distance involving the methyl carbon was restrained to 1.50±0.01 Å and the 1,3-related ones to 2.50±0.01 Å. The anisotropic displacement parameters of the seven carbon atoms were restrained to be nearly isotropic.

The iminium and hydroxy H-atoms were located in a difference Fourier map and they were freely refined.

### Crystal data

**Table:**

C_30_H_33_Cl_3_N_4_O_3_·0.5C_7_H_8_	*Z* = 2
*M**_r_* = 650.02	*F*(000) = 682
Triclinic, *P*1	*D*_x_ = 1.357 Mg m^−^^3^
Hall symbol: -P 1	Mo *K*α radiation, λ = 0.71073 Å
*a* = 7.3651 (2) Å	Cell parameters from 3243 reflections
*b* = 11.1839 (2) Å	θ = 2.3–29.3°
*c* = 20.1594 (5) Å	µ = 0.33 mm^−^^1^
α = 100.618 (2)°	*T* = 100 K
β = 97.765 (2)°	Plate, yellow
γ = 98.560 (2)°	0.22 × 0.18 × 0.02 mm
*V* = 1591.20 (7) Å^3^	

### Data collection

**Table:**

Bruker SMART APEX diffractometer	7269 independent reflections
Radiation source: fine-focus sealed tube	4901 reflections with *I* > 2σ(*I*)
graphite	*R*_int_ = 0.033
ω scans	θ_max_ = 27.5°, θ_min_ = 1.0°
Absorption correction: multi-scan (*SADABS*; Sheldrick, 1996)	*h* = −9→9
*T*_min_ = 0.931, *T*_max_ = 0.993	*k* = −14→14
14959 measured reflections	*l* = −26→25

### Refinement

**Table:**

Refinement on *F*^2^	Primary atom site location: structure-invariant direct methods
Least-squares matrix: full	Secondary atom site location: difference Fourier map
*R*[*F*^2^ > 2σ(*F*^2^)] = 0.050	Hydrogen site location: inferred from neighbouring sites
*wR*(*F*^2^) = 0.150	H atoms treated by a mixture of independent and constrained refinement
*S* = 1.04	*w* = 1/[σ^2^(*F*_o_^2^) + (0.0807*P*)^2^ + 0.0533*P*] where *P* = (*F*_o_^2^ + 2*F*_c_^2^)/3
7269 reflections	(Δ/σ)_max_ = 0.001
410 parameters	Δρ_max_ = 0.79 e Å^−^^3^
30 restraints	Δρ_min_ = −0.80 e Å^−^^3^

### Fractional atomic coordinates and isotropic or equivalent isotropic displacement parameters (Å^2^)

**Table:**

	*x*	*y*	*z*	*U*_iso_*/*U*_eq_	Occ. (<1)
Cl1	−0.23379 (11)	0.63575 (6)	0.19284 (4)	0.03291 (19)	
Cl2	0.23675 (10)	0.10135 (7)	0.68824 (3)	0.03301 (19)	
Cl3	0.21656 (11)	0.59383 (6)	0.09250 (5)	0.0398 (2)	
O1	0.0640 (3)	0.21520 (18)	0.27035 (9)	0.0250 (4)	
O2	0.1122 (3)	−0.23059 (17)	0.41888 (9)	0.0268 (4)	
O3	0.4300 (2)	0.11432 (15)	0.00308 (9)	0.0209 (4)	
N1	0.0135 (3)	0.11221 (18)	0.14742 (10)	0.0173 (4)	
N2	0.2570 (3)	−0.05363 (19)	0.37189 (10)	0.0203 (5)	
N3	0.5052 (3)	0.0992 (2)	0.12427 (11)	0.0194 (4)	
N4	0.2407 (3)	−0.07266 (19)	0.18467 (10)	0.0185 (4)	
C1	0.0036 (3)	0.3111 (2)	0.25116 (13)	0.0202 (5)	
C2	−0.0030 (4)	0.4161 (3)	0.30111 (13)	0.0263 (6)	
H2	0.0400	0.4174	0.3479	0.032*	
C3	−0.0698 (4)	0.5162 (2)	0.28386 (14)	0.0267 (6)	
H3	−0.0713	0.5867	0.3182	0.032*	
C4	−0.1358 (4)	0.5134 (2)	0.21528 (13)	0.0229 (6)	
C5	−0.1268 (3)	0.4145 (2)	0.16472 (13)	0.0196 (5)	
H5	−0.1684	0.4160	0.1182	0.024*	
C6	−0.0570 (3)	0.3114 (2)	0.18097 (12)	0.0172 (5)	
C7	−0.0454 (3)	0.2053 (2)	0.12730 (12)	0.0159 (5)	
C8	−0.1005 (3)	0.2054 (2)	0.05297 (12)	0.0199 (5)	
H8A	0.0064	0.1964	0.0295	0.030*	
H8B	−0.1410	0.2834	0.0483	0.030*	
H8C	−0.2028	0.1363	0.0325	0.030*	
C9	0.0297 (3)	0.0003 (2)	0.09993 (13)	0.0185 (5)	
H9A	0.1345	0.0184	0.0752	0.022*	
H9B	−0.0860	−0.0278	0.0658	0.022*	
C10	0.0625 (3)	−0.1009 (2)	0.13845 (13)	0.0190 (5)	
H10A	−0.0391	−0.1145	0.1652	0.023*	
H10B	0.0573	−0.1787	0.1050	0.023*	
C11	0.1462 (3)	−0.1520 (2)	0.47997 (13)	0.0210 (5)	
C12	0.0918 (4)	−0.1964 (3)	0.53595 (13)	0.0247 (6)	
H12	0.0335	−0.2803	0.5298	0.030*	
C13	0.1212 (4)	−0.1205 (3)	0.60011 (13)	0.0260 (6)	
H13	0.0837	−0.1516	0.6379	0.031*	
C14	0.2062 (3)	0.0015 (3)	0.60846 (13)	0.0238 (6)	
C15	0.2619 (3)	0.0482 (2)	0.55447 (13)	0.0215 (5)	
H15	0.3199	0.1323	0.5617	0.026*	
C16	0.2337 (3)	−0.0273 (2)	0.48890 (12)	0.0188 (5)	
C17	0.2881 (3)	0.0227 (2)	0.43020 (12)	0.0193 (5)	
C18	0.3728 (4)	0.1565 (2)	0.43895 (14)	0.0266 (6)	
H18A	0.3127	0.1905	0.4020	0.040*	
H18B	0.5064	0.1639	0.4374	0.040*	
H18C	0.3546	0.2025	0.4832	0.040*	
C19	0.2943 (4)	−0.0111 (2)	0.30971 (12)	0.0241 (6)	
H19A	0.4289	0.0214	0.3141	0.029*	
H19B	0.2253	0.0566	0.3034	0.029*	
C20	0.2347 (4)	−0.1166 (3)	0.24873 (13)	0.0256 (6)	
H20A	0.3182	−0.1778	0.2515	0.031*	
H20B	0.1065	−0.1581	0.2491	0.031*	
C21	0.3929 (3)	0.2251 (2)	0.02511 (13)	0.0191 (5)	
C22	0.3371 (3)	0.2955 (2)	−0.02214 (14)	0.0221 (5)	
H22	0.3345	0.2646	−0.0695	0.027*	
C23	0.2860 (4)	0.4080 (2)	−0.00169 (15)	0.0258 (6)	
H23	0.2471	0.4539	−0.0346	0.031*	
C24	0.2918 (4)	0.4542 (2)	0.06778 (15)	0.0251 (6)	
C25	0.3537 (3)	0.3918 (2)	0.11611 (14)	0.0238 (6)	
H25	0.3616	0.4269	0.1633	0.029*	
C26	0.4060 (3)	0.2759 (2)	0.09650 (13)	0.0190 (5)	
C27	0.4692 (3)	0.2072 (2)	0.14771 (13)	0.0203 (5)	
C28	0.4898 (4)	0.2621 (3)	0.22289 (13)	0.0263 (6)	
H28A	0.5594	0.2133	0.2493	0.040*	
H28B	0.3662	0.2612	0.2359	0.040*	
H28C	0.5572	0.3474	0.2324	0.040*	
C29	0.5577 (3)	0.0091 (2)	0.16366 (14)	0.0249 (6)	
H29A	0.5869	0.0481	0.2130	0.030*	
H29B	0.6703	−0.0197	0.1498	0.030*	
C30	0.3992 (3)	−0.1001 (2)	0.15147 (13)	0.0197 (5)	
H30A	0.3555	−0.1282	0.1016	0.024*	
H30B	0.4468	−0.1689	0.1685	0.024*	
C31	0.4925 (19)	0.5285 (12)	0.4490 (5)	0.083 (3)	0.50
C32	0.3426 (19)	0.5415 (12)	0.4832 (5)	0.059 (3)	0.50
H32	0.2419	0.5754	0.4641	0.071*	0.50
C33	0.340 (2)	0.5050 (13)	0.5454 (5)	0.089 (3)	0.50
H33	0.2376	0.5139	0.5688	0.107*	0.50
C34	0.487 (2)	0.4554 (12)	0.5734 (5)	0.083 (3)	0.50
H34	0.4856	0.4305	0.6159	0.100*	0.50
C35	0.637 (2)	0.4424 (12)	0.5392 (5)	0.059 (3)	0.50
H35	0.7379	0.4085	0.5584	0.071*	0.50
C36	0.6399 (19)	0.4789 (12)	0.4770 (5)	0.089 (3)	0.50
H36	0.7423	0.4700	0.4537	0.107*	0.50
C37	0.5056 (18)	0.5705 (11)	0.3829 (5)	0.123 (5)	0.50
H37A	0.4289	0.6342	0.3792	0.185*	0.50
H37B	0.4608	0.5000	0.3444	0.185*	0.50
H37C	0.6355	0.6046	0.3820	0.185*	0.50
H1O	0.055 (7)	0.163 (4)	0.2335 (15)	0.109 (19)*	
H2O	0.150 (5)	−0.183 (3)	0.3934 (17)	0.070 (13)*	
H3N	0.484 (7)	0.086 (5)	0.0787 (6)	0.106 (17)*	

### Atomic displacement parameters (Å^2^)

**Table:**

	*U*^11^	*U*^22^	*U*^33^	*U*^12^	*U*^13^	*U*^23^
Cl1	0.0467 (4)	0.0175 (3)	0.0363 (4)	0.0104 (3)	0.0104 (3)	0.0038 (3)
Cl2	0.0319 (4)	0.0504 (4)	0.0164 (3)	0.0158 (3)	0.0031 (3)	0.0000 (3)
Cl3	0.0402 (4)	0.0188 (3)	0.0650 (5)	0.0116 (3)	0.0178 (4)	0.0090 (3)
O1	0.0271 (10)	0.0307 (10)	0.0202 (10)	0.0102 (8)	0.0048 (8)	0.0083 (8)
O2	0.0335 (11)	0.0250 (10)	0.0218 (10)	0.0038 (9)	0.0068 (8)	0.0040 (8)
O3	0.0219 (9)	0.0183 (8)	0.0240 (9)	0.0072 (7)	0.0046 (7)	0.0047 (7)
N1	0.0129 (10)	0.0182 (10)	0.0211 (11)	0.0020 (8)	0.0049 (8)	0.0042 (8)
N2	0.0199 (11)	0.0250 (11)	0.0180 (11)	0.0057 (9)	0.0054 (8)	0.0066 (9)
N3	0.0152 (10)	0.0222 (11)	0.0229 (12)	0.0020 (8)	0.0034 (9)	0.0106 (9)
N4	0.0176 (10)	0.0238 (11)	0.0159 (10)	0.0042 (9)	0.0049 (8)	0.0073 (8)
C1	0.0168 (12)	0.0246 (13)	0.0192 (12)	0.0012 (10)	0.0055 (10)	0.0044 (10)
C2	0.0269 (14)	0.0314 (14)	0.0178 (13)	0.0006 (12)	0.0041 (11)	0.0010 (11)
C3	0.0286 (14)	0.0230 (13)	0.0242 (14)	0.0005 (11)	0.0067 (11)	−0.0043 (11)
C4	0.0230 (13)	0.0178 (12)	0.0278 (14)	0.0010 (10)	0.0072 (11)	0.0040 (11)
C5	0.0197 (12)	0.0185 (12)	0.0202 (13)	0.0009 (10)	0.0037 (10)	0.0050 (10)
C6	0.0143 (11)	0.0195 (12)	0.0181 (12)	0.0005 (10)	0.0043 (9)	0.0051 (10)
C7	0.0112 (11)	0.0184 (12)	0.0185 (12)	0.0013 (9)	0.0045 (9)	0.0045 (10)
C8	0.0213 (13)	0.0206 (12)	0.0194 (12)	0.0061 (10)	0.0051 (10)	0.0053 (10)
C9	0.0155 (12)	0.0184 (12)	0.0217 (13)	0.0042 (10)	0.0030 (10)	0.0037 (10)
C10	0.0134 (11)	0.0209 (12)	0.0233 (13)	0.0023 (10)	0.0043 (10)	0.0060 (10)
C11	0.0191 (12)	0.0276 (13)	0.0184 (13)	0.0084 (11)	0.0043 (10)	0.0058 (10)
C12	0.0204 (13)	0.0308 (14)	0.0264 (14)	0.0083 (11)	0.0045 (11)	0.0115 (12)
C13	0.0217 (13)	0.0415 (16)	0.0207 (13)	0.0128 (12)	0.0078 (11)	0.0126 (12)
C14	0.0189 (13)	0.0384 (15)	0.0149 (12)	0.0127 (12)	0.0015 (10)	0.0020 (11)
C15	0.0177 (12)	0.0250 (13)	0.0223 (13)	0.0089 (11)	0.0006 (10)	0.0042 (11)
C16	0.0132 (12)	0.0255 (13)	0.0190 (12)	0.0074 (10)	0.0023 (9)	0.0051 (10)
C17	0.0135 (11)	0.0257 (13)	0.0200 (13)	0.0071 (10)	0.0024 (10)	0.0055 (10)
C18	0.0282 (14)	0.0257 (14)	0.0253 (14)	0.0020 (12)	0.0060 (11)	0.0048 (11)
C19	0.0276 (14)	0.0272 (14)	0.0189 (13)	0.0047 (11)	0.0072 (11)	0.0066 (11)
C20	0.0301 (15)	0.0285 (14)	0.0197 (13)	0.0024 (12)	0.0073 (11)	0.0091 (11)
C21	0.0115 (11)	0.0171 (12)	0.0299 (14)	0.0016 (9)	0.0054 (10)	0.0072 (10)
C22	0.0179 (12)	0.0243 (13)	0.0259 (14)	0.0033 (10)	0.0057 (10)	0.0082 (11)
C23	0.0179 (13)	0.0220 (13)	0.0413 (16)	0.0020 (10)	0.0072 (12)	0.0158 (12)
C24	0.0203 (13)	0.0141 (12)	0.0424 (16)	0.0022 (10)	0.0106 (12)	0.0062 (11)
C25	0.0177 (13)	0.0195 (12)	0.0320 (15)	−0.0008 (10)	0.0073 (11)	0.0012 (11)
C26	0.0134 (11)	0.0192 (12)	0.0240 (13)	−0.0009 (10)	0.0043 (10)	0.0053 (10)
C27	0.0118 (11)	0.0233 (13)	0.0246 (13)	−0.0031 (10)	0.0051 (10)	0.0051 (11)
C28	0.0243 (14)	0.0301 (14)	0.0234 (14)	−0.0007 (12)	0.0069 (11)	0.0050 (11)
C29	0.0160 (12)	0.0314 (14)	0.0315 (15)	0.0048 (11)	0.0032 (11)	0.0172 (12)
C30	0.0166 (12)	0.0226 (12)	0.0241 (13)	0.0080 (10)	0.0057 (10)	0.0102 (10)
C31	0.112 (5)	0.036 (4)	0.081 (7)	0.007 (3)	−0.021 (5)	−0.009 (4)
C32	0.058 (3)	0.036 (3)	0.072 (6)	0.019 (3)	−0.013 (4)	−0.008 (4)
C33	0.090 (4)	0.050 (4)	0.109 (7)	0.023 (3)	−0.030 (5)	−0.009 (5)
C34	0.112 (5)	0.036 (4)	0.081 (7)	0.007 (3)	−0.021 (5)	−0.009 (4)
C35	0.058 (3)	0.036 (3)	0.072 (6)	0.019 (3)	−0.013 (4)	−0.008 (4)
C36	0.090 (4)	0.050 (4)	0.109 (7)	0.023 (3)	−0.030 (5)	−0.009 (5)
C37	0.142 (8)	0.097 (7)	0.118 (8)	−0.017 (6)	0.060 (7)	−0.009 (6)

### Geometric parameters (Å, °)

**Table:**

Cl1—C4	1.742 (3)	C15—H15	0.9500
Cl2—C14	1.746 (3)	C16—C17	1.476 (3)
Cl3—C24	1.744 (3)	C17—C18	1.502 (4)
O1—C1	1.321 (3)	C18—H18A	0.9800
O1—H1O	0.846 (10)	C18—H18B	0.9800
O2—C11	1.343 (3)	C18—H18C	0.9800
O2—H2O	0.846 (10)	C19—C20	1.504 (4)
O3—C21	1.318 (3)	C19—H19A	0.9900
N1—C7	1.296 (3)	C19—H19B	0.9900
N1—C9	1.459 (3)	C20—H20A	0.9900
N2—C17	1.289 (3)	C20—H20B	0.9900
N2—C19	1.465 (3)	C21—C22	1.401 (4)
N3—C27	1.293 (3)	C21—C26	1.431 (4)
N3—C29	1.457 (3)	C22—C23	1.374 (4)
N3—H3N	0.892 (10)	C22—H22	0.9500
N4—C10	1.459 (3)	C23—C24	1.393 (4)
N4—C30	1.465 (3)	C23—H23	0.9500
N4—C20	1.468 (3)	C24—C25	1.367 (4)
C1—C2	1.411 (4)	C25—C26	1.409 (3)
C1—C6	1.426 (3)	C25—H25	0.9500
C2—C3	1.371 (4)	C26—C27	1.463 (4)
C2—H2	0.9500	C27—C28	1.505 (4)
C3—C4	1.395 (4)	C28—H28A	0.9800
C3—H3	0.9500	C28—H28B	0.9800
C4—C5	1.375 (3)	C28—H28C	0.9800
C5—C6	1.405 (3)	C29—C30	1.517 (4)
C5—H5	0.9500	C29—H29A	0.9900
C6—C7	1.472 (3)	C29—H29B	0.9900
C7—C8	1.499 (3)	C30—H30A	0.9900
C8—H8A	0.9800	C30—H30B	0.9900
C8—H8B	0.9800	C31—C32	1.3900
C8—H8C	0.9800	C31—C36	1.3900
C9—C10	1.516 (3)	C31—C37	1.503 (6)
C9—H9A	0.9900	C32—C33	1.3900
C9—H9B	0.9900	C32—H32	0.9500
C10—H10A	0.9900	C33—C34	1.3900
C10—H10B	0.9900	C33—H33	0.9500
C11—C12	1.397 (4)	C34—C35	1.3900
C11—C16	1.414 (4)	C34—H34	0.9500
C12—C13	1.380 (4)	C35—C36	1.3900
C12—H12	0.9500	C35—H35	0.9500
C13—C14	1.384 (4)	C36—H36	0.9500
C13—H13	0.9500	C37—H37A	0.9800
C14—C15	1.375 (4)	C37—H37B	0.9800
C15—C16	1.402 (3)	C37—H37C	0.9800
			
C1—O1—H1O	105 (3)	C17—C18—H18C	109.5
C11—O2—H2O	101 (3)	H18A—C18—H18C	109.5
C7—N1—C9	122.8 (2)	H18B—C18—H18C	109.5
C17—N2—C19	120.7 (2)	N2—C19—C20	109.7 (2)
C27—N3—C29	127.2 (2)	N2—C19—H19A	109.7
C27—N3—H3N	108 (3)	C20—C19—H19A	109.7
C29—N3—H3N	124 (3)	N2—C19—H19B	109.7
C10—N4—C30	114.31 (19)	C20—C19—H19B	109.7
C10—N4—C20	114.1 (2)	H19A—C19—H19B	108.2
C30—N4—C20	114.0 (2)	N4—C20—C19	110.9 (2)
O1—C1—C2	119.6 (2)	N4—C20—H20A	109.4
O1—C1—C6	121.8 (2)	C19—C20—H20A	109.4
C2—C1—C6	118.6 (2)	N4—C20—H20B	109.4
C3—C2—C1	121.7 (2)	C19—C20—H20B	109.4
C3—C2—H2	119.2	H20A—C20—H20B	108.0
C1—C2—H2	119.2	O3—C21—C22	119.7 (2)
C2—C3—C4	119.1 (2)	O3—C21—C26	121.8 (2)
C2—C3—H3	120.4	C22—C21—C26	118.5 (2)
C4—C3—H3	120.4	C23—C22—C21	121.6 (2)
C5—C4—C3	121.1 (2)	C23—C22—H22	119.2
C5—C4—Cl1	119.0 (2)	C21—C22—H22	119.2
C3—C4—Cl1	119.8 (2)	C22—C23—C24	119.3 (3)
C4—C5—C6	120.8 (2)	C22—C23—H23	120.4
C4—C5—H5	119.6	C24—C23—H23	120.4
C6—C5—H5	119.6	C25—C24—C23	121.3 (2)
C5—C6—C1	118.5 (2)	C25—C24—Cl3	120.1 (2)
C5—C6—C7	121.4 (2)	C23—C24—Cl3	118.6 (2)
C1—C6—C7	120.1 (2)	C24—C25—C26	120.5 (2)
N1—C7—C6	116.9 (2)	C24—C25—H25	119.7
N1—C7—C8	121.8 (2)	C26—C25—H25	119.7
C6—C7—C8	121.3 (2)	C25—C26—C21	118.7 (2)
C7—C8—H8A	109.5	C25—C26—C27	121.0 (2)
C7—C8—H8B	109.5	C21—C26—C27	120.3 (2)
H8A—C8—H8B	109.5	N3—C27—C26	116.1 (2)
C7—C8—H8C	109.5	N3—C27—C28	123.3 (2)
H8A—C8—H8C	109.5	C26—C27—C28	120.6 (2)
H8B—C8—H8C	109.5	C27—C28—H28A	109.5
N1—C9—C10	110.2 (2)	C27—C28—H28B	109.5
N1—C9—H9A	109.6	H28A—C28—H28B	109.5
C10—C9—H9A	109.6	C27—C28—H28C	109.5
N1—C9—H9B	109.6	H28A—C28—H28C	109.5
C10—C9—H9B	109.6	H28B—C28—H28C	109.5
H9A—C9—H9B	108.1	N3—C29—C30	109.8 (2)
N4—C10—C9	113.3 (2)	N3—C29—H29A	109.7
N4—C10—H10A	108.9	C30—C29—H29A	109.7
C9—C10—H10A	108.9	N3—C29—H29B	109.7
N4—C10—H10B	108.9	C30—C29—H29B	109.7
C9—C10—H10B	108.9	H29A—C29—H29B	108.2
H10A—C10—H10B	107.7	N4—C30—C29	113.8 (2)
O2—C11—C12	118.0 (2)	N4—C30—H30A	108.8
O2—C11—C16	122.3 (2)	C29—C30—H30A	108.8
C12—C11—C16	119.7 (2)	N4—C30—H30B	108.8
C13—C12—C11	121.1 (3)	C29—C30—H30B	108.8
C13—C12—H12	119.4	H30A—C30—H30B	107.7
C11—C12—H12	119.4	C32—C31—C36	120.0
C12—C13—C14	118.9 (2)	C32—C31—C37	122.2 (5)
C12—C13—H13	120.6	C36—C31—C37	117.8 (5)
C14—C13—H13	120.6	C33—C32—C31	120.0
C15—C14—C13	121.5 (2)	C33—C32—H32	120.0
C15—C14—Cl2	118.5 (2)	C31—C32—H32	120.0
C13—C14—Cl2	119.9 (2)	C32—C33—C34	120.0
C14—C15—C16	120.6 (2)	C32—C33—H33	120.0
C14—C15—H15	119.7	C34—C33—H33	120.0
C16—C15—H15	119.7	C33—C34—C35	120.0
C15—C16—C11	118.2 (2)	C33—C34—H34	120.0
C15—C16—C17	121.0 (2)	C35—C34—H34	120.0
C11—C16—C17	120.7 (2)	C36—C35—C34	120.0
N2—C17—C16	116.6 (2)	C36—C35—H35	120.0
N2—C17—C18	122.5 (2)	C34—C35—H35	120.0
C16—C17—C18	120.8 (2)	C35—C36—C31	120.0
C17—C18—H18A	109.5	C35—C36—H36	120.0
C17—C18—H18B	109.5	C31—C36—H36	120.0
H18A—C18—H18B	109.5		
			
O1—C1—C2—C3	−178.0 (2)	C11—C16—C17—N2	−2.1 (3)
C6—C1—C2—C3	1.6 (4)	C15—C16—C17—C18	−0.9 (3)
C1—C2—C3—C4	1.0 (4)	C11—C16—C17—C18	177.1 (2)
C2—C3—C4—C5	−3.0 (4)	C17—N2—C19—C20	−176.4 (2)
C2—C3—C4—Cl1	176.2 (2)	C10—N4—C20—C19	−121.4 (2)
C3—C4—C5—C6	2.3 (4)	C30—N4—C20—C19	104.7 (2)
Cl1—C4—C5—C6	−176.95 (19)	N2—C19—C20—N4	170.4 (2)
C4—C5—C6—C1	0.4 (4)	O3—C21—C22—C23	175.9 (2)
C4—C5—C6—C7	−179.5 (2)	C26—C21—C22—C23	−3.4 (4)
O1—C1—C6—C5	177.3 (2)	C21—C22—C23—C24	0.8 (4)
C2—C1—C6—C5	−2.2 (3)	C22—C23—C24—C25	2.4 (4)
O1—C1—C6—C7	−2.8 (4)	C22—C23—C24—Cl3	−177.2 (2)
C2—C1—C6—C7	177.7 (2)	C23—C24—C25—C26	−2.8 (4)
C9—N1—C7—C6	179.1 (2)	Cl3—C24—C25—C26	176.78 (19)
C9—N1—C7—C8	−0.5 (3)	C24—C25—C26—C21	0.0 (4)
C5—C6—C7—N1	−177.2 (2)	C24—C25—C26—C27	−178.9 (2)
C1—C6—C7—N1	2.9 (3)	O3—C21—C26—C25	−176.3 (2)
C5—C6—C7—C8	2.5 (3)	C22—C21—C26—C25	3.0 (3)
C1—C6—C7—C8	−177.4 (2)	O3—C21—C26—C27	2.6 (3)
C7—N1—C9—C10	−168.1 (2)	C22—C21—C26—C27	−178.1 (2)
C30—N4—C10—C9	−82.6 (2)	C29—N3—C27—C26	−175.5 (2)
C20—N4—C10—C9	143.6 (2)	C29—N3—C27—C28	4.6 (4)
N1—C9—C10—N4	−65.6 (3)	C25—C26—C27—N3	176.8 (2)
O2—C11—C12—C13	−179.5 (2)	C21—C26—C27—N3	−2.2 (3)
C16—C11—C12—C13	0.2 (4)	C25—C26—C27—C28	−3.3 (3)
C11—C12—C13—C14	0.0 (4)	C21—C26—C27—C28	177.8 (2)
C12—C13—C14—C15	−0.1 (4)	C27—N3—C29—C30	109.5 (3)
C12—C13—C14—Cl2	177.42 (19)	C10—N4—C30—C29	125.5 (2)
C13—C14—C15—C16	0.0 (4)	C20—N4—C30—C29	−100.7 (2)
Cl2—C14—C15—C16	−177.57 (18)	N3—C29—C30—N4	−72.8 (3)
C14—C15—C16—C11	0.2 (3)	C36—C31—C32—C33	0.0
C14—C15—C16—C17	178.3 (2)	C37—C31—C32—C33	−177.9 (9)
O2—C11—C16—C15	179.4 (2)	C31—C32—C33—C34	0.0
C12—C11—C16—C15	−0.4 (3)	C32—C33—C34—C35	0.0
O2—C11—C16—C17	1.4 (4)	C33—C34—C35—C36	0.0
C12—C11—C16—C17	−178.4 (2)	C34—C35—C36—C31	0.0
C19—N2—C17—C16	175.8 (2)	C32—C31—C36—C35	0.0
C19—N2—C17—C18	−3.4 (4)	C37—C31—C36—C35	177.9 (8)
C15—C16—C17—N2	179.9 (2)		

### Hydrogen-bond geometry (Å, °)

**Table:**

*D*—H···*A*	*D*—H	H···*A*	*D*···*A*	*D*—H···*A*
O1—H1o···N1	0.85 (1)	1.69 (2)	2.486 (3)	155 (5)
O2—H2o···N2	0.85 (1)	1.70 (2)	2.507 (3)	158 (4)
N3—H3n···O3	0.89 (1)	1.62 (2)	2.474 (3)	158 (5)
